# Parasitic nematode *Meloidogyne incognita* interactions with different *Capsicum annum* cultivars reveal the chemical constituents modulating root herbivory

**DOI:** 10.1038/s41598-017-02379-8

**Published:** 2017-06-06

**Authors:** Ruth Kihika, Lucy K. Murungi, Danny Coyne, Margaret Ng’ang’a, Ahmed Hassanali, Peter E. A. Teal, Baldwyn Torto

**Affiliations:** 1 Behavioural and Chemical Ecology Unit, International Centre of Insect Physiology and Ecology, P.O. Box 30772-00100 Nairobi, Kenya; 2 Kenyatta University, P.O. Box 43844- 00100 Nairobi, Kenya; 3 Jomo Kenyatta University of Agriculture and Technology, P.O. Box 62000-00200 Nairobi, Kenya; 4 International Institute of Tropical Agriculture (IITA), P.O. Box 30772-00100 Nairobi, Kenya; 5 USDA/ARS-CMAVE, 1600/1700 SW23rd Dr., Gainesville, FL 32608 USA

## Abstract

Plant volatile signatures are often used as cues by herbivores to locate their preferred hosts. Here, we report on the volatile organic compounds used by the subterranean root-knot nematode (RKN) *Meloidogyne incognita* for host location. We compared responses of infective second stage juveniles (J2s) to root volatiles of three cultivars and one accession of the solanaceous plant, *Capsicum annum* against moist sand in dual choice assays. J2s were more attracted to the three cultivars than to the accession, relative to controls. GC/MS analysis of the volatiles identified common constituents in each plant, five of which were identified as α-pinene, limonene, 2-methoxy-3-(1-methylpropyl)-pyrazine, methyl salicylate and tridecane. We additionally identified thymol as being specific to the accession. In dose-response assays, a blend of the five components elicited positive chemotaxis (71–88%), whereas individual components elicited varying responses; Methyl salicylate (MeSA) elicited the highest positive chemotaxis (70–80%), α-pinene, limonene and tridecane were intermediate (54–60%), and 2-methoxy-3-(1-methylpropyl)-pyrazine the lowest (49–55%). In contrast, thymol alone or thymol combined with either the preferred natural plant root volatiles or the five-component synthetic blend induced negative chemotaxis. Our results provide insights into RKN-host plant interactions, creating new opportunities for plant breeding programmes towards management of RKNs.

## Introduction

Plants have evolved in diverse ecologies with complex interactions with other organisms such as microbes and arthropods. Most of these interactions are mediated by chemical signals. Plant derived volatile organic compounds (VOCs) mediate above- and below-ground interactions and their potential use to manipulate herbivore behavior has been demonstrated in several studies^1–9^. Below ground interactions are important because roots constitute the primary pathway of plant nutrient and water acquisition for successful growth and development. Previous work on below-ground interactions has shown that herbivore-induced plant volatiles (HIPVs) in the subterranean environment benefit plants by recruiting natural enemies of herbivorous insects. For example, (*E*)-β-caryophyllene, a maize produced HIPV is a specific recruitment signal for the entomopathogenic nematode (EPN) *Heterorhabditis megidis* Poinar, Jackson & Klein in response to feeding by the beetle *Diabrotica virgifera virgifera* ^10^. Similarly, pregeijerene is released by citrus (*Citrus paradisi* Macf. x *Poncirus trifoliata* L. Raf.) root stocks infested by larvae of the weevil *Diaprepes abbreviates* (L.) and attracts a variety of EPN species^11, 12^. Other than recruiting EPNs, plant produced volatiles have been shown to modulate EPNs inter-specific social behavioral plasticity, learning, and memory^13^. Compared to EPNs, responses of plant parasitic nematodes (PPNs) to plant volatiles has received little attention. In one study, the citrus sedentary root nematode, *Tylenchulus semipenetrans* Cobb, was shown to be attracted to weevil infested citrus roots compared to uninfested plants^14^. Economically important PPNs such as the root-knot nematode (RKNs; *Meloidogyne* spp.) remain largely uninvestigated in their response to plant volatiles.

Nematodes of the genus *Meloidogyne* are sedentary endoparasitic PPNs that are highly polyphagous and cause damage to a wide range of economically important crops worldwide^15^. In Africa, infection results in up to 100% yield losses particularly in high value vegetables^16, 17^. The infective stage is the second juvenile stage (J2) that infects the vascular bundles of plants and converts them into metabolically active ‘giant cells’ that are exploited for nutrients^16, 18^. In the process, nutrient and water uptake by the host plant is impeded resulting in poor growth and crop yield^16^. Additionally, such damage increases the severity of opportunistic infections from other soil pathogens^19^. The southern RKN, *Meloidogyne incognita* Kofoid and White (Chitwood), is one of the most damaging species due to its broad host range and high rate of reproduction. *M*. *incognita* has a high propensity for solanaceous crops, such as tomato, pepper and African Leafy Vegetables (ALV)^16, 18, 20, 21^. Several methods including crop rotation, resistant cultivars, biological control and nematicides are used to control *M*. *incognita* and other RKNs^18, 22^. Of the existing methods, fumigant nematicides such as methyl bromide were most promising, but due to their ozone-depleting properties their use has been completely phased-out^16^ necessitating alternative ecofriendly control strategies.

One of such approaches would be to use the chemical cues involved in the host plant-RKN interaction. Knowledge of chemical communication in plant-pest interactions has had resounding success in developing technologies for pest control. A good example is the ‘push-pull’ technology developed for stem borer and striga management^23, 24^. For RKNs, a potential target area – or weak link would be host location by J2s that is chemically mediated. The J2s detect VOCs produced by the roots of the host plants or by associated rhizosphere micro-organisms^25–27^ using a combination of head and tail chemosensory organs^16, 18, 28^. Although olfaction is considered to be fundamental in host seeking, identity of the specific olfactory cues remain unknown. Carbon dioxide (CO_2_) has previously been demonstrated to be a generalist attractant for nematodes^29^ but, a recent study indicates that it may serve as a response enhancer to more specific chemical cues^30^.

To elucidate the semiochemical basis of RKN host location, we used bioassay-guided chemical analysis to identify root constituents of different pepper plants that mediate the host finding behavior of RKNs. We selected pepper (*Capsicum* species), in part because of its high economic value as a globally cultivated vegetable crop^31^ in addition to RKNs being an important limiting factor in their production^32–34^. Three pepper cultivars, California Wonder, Yolo Wonder and Long Red Cayenne, commonly grown in small- and large-scale production systems in East Africa, and one accession, AVDRC PP0237 that are differentially attacked by RKNs, were used. We hypothesized that volatiles produced by the roots of these pepper plants may influence the host seeking behavior of RKNs. To test this hypothesis we: (i) assessed the rate of RKN root infection using the galling and egg mass indices described by Taylor & Sasser^22^, (ii) investigated the responses of *M*. *incognita* to root volatiles of the pepper plants, (iii) compared their root VOC profiles, (iv) identified the constituents associated with any differential responses of the nematodes to the pepper plants.

## Results

### *Meloidogyne incognita* infestation on pepper cultivars

Galling index is a measure for assessing RKN infection on a plant by counting the number of galls per root system. Egg mass index assesses the reproduction of the nematodes and can be used as a measure for susceptibility or resistance of plants to RKNs^16, 22^. Assessment of the incidence of root-knot disease showed that cultivars California Wonder, Yolo Wonder and Long Red Cayenne recorded 2–4 times more galling and egg mass indices than for the accession AVDRC PP0237 (Table 1).

**Table 1:** Galling and egg-mass indices of California Wonder, Yolo Wonder, Long Red Cayenne and accession AVDRC PP0237.

Pepper plant	No. of galls^Ŧ^	Galling index	Egg masses^Ŧ^	Egg mass index
California Wonder	44.72^a^	4.00^a^	37.27^a^	3.75^a^
Yolo Wonder	32.69^b^	3.75^ab^	35.57^a^	3.50^a^
Long Red Cayenne	22.42^b^	3.00^b^	25.07^a^	3.00^a^
AVDRC PP0237	0.75^c^	0.75^c^	0.00^b^	0.00^b^

^Ŧ^Mean number of galls and egg masses per root system of four replicates. Each pot containing five plants was considered as a replicate. Means with different letters in the same column are significantly different (P < 0.05, Duncan’s Multiple Range test). Galling and egg mass indices were done using the scale: 0 = no galls or no egg masses, 1 = 1 to 2, 2 = 3 to 10, 3 = 11 to 30, 4 = 31 to 100, and 5 = more than 100 galls or more than 100 egg masses per plant^22, 54^.

### Root volatiles of *Capsicum annum* modulate responses of *Meloidogyne incognita*

**Figure 1:**
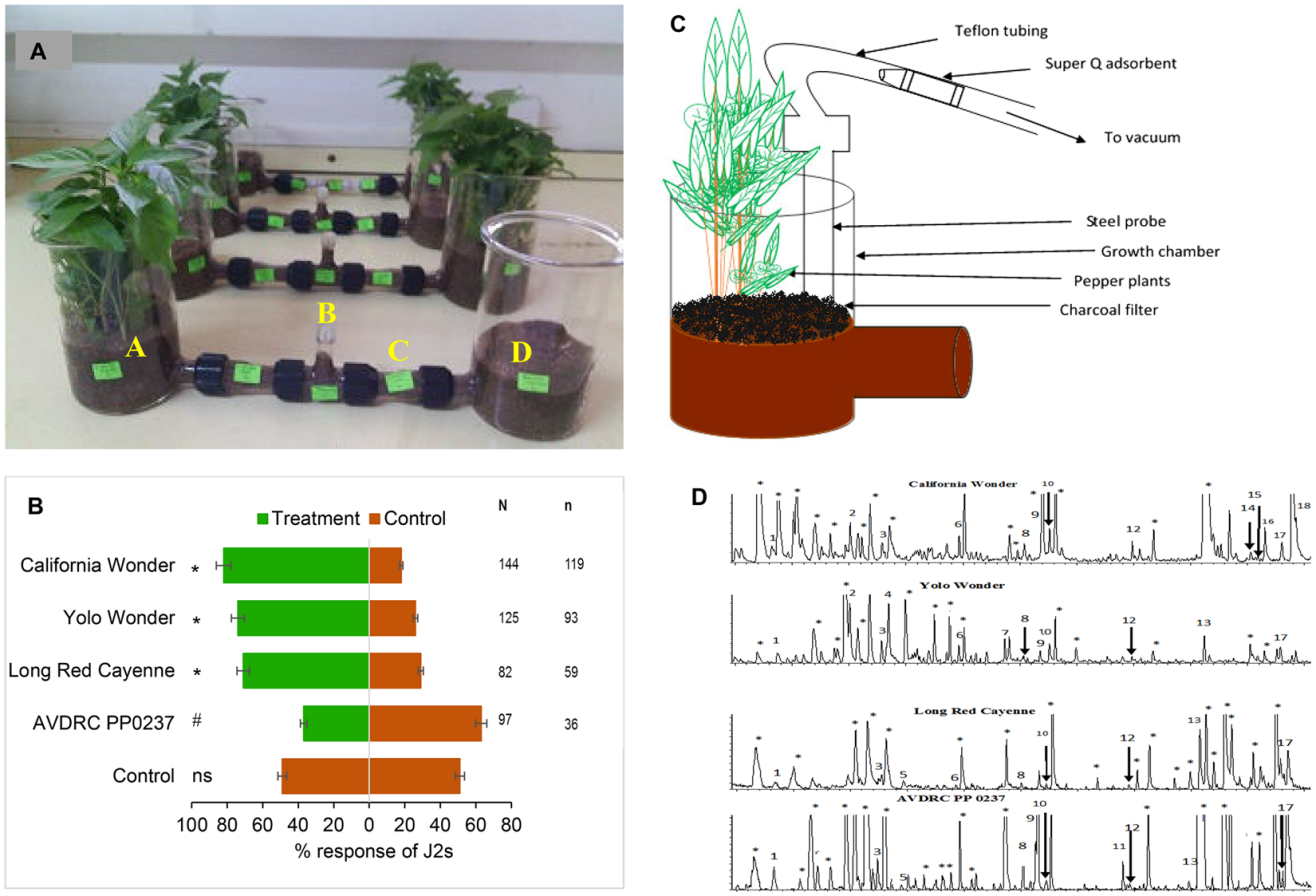
Response of *Meloidogyne incognita* infective juveniles (J2s) to root volatiles of pepper and chemical analysis of the root volatiles. (**A**) Dual choice olfactometer assays to test J2 responses to *Capsicum annum* root volatiles and synthetic blends: (**A**) Stimulus chamber, (**B**) Release arm (**C**) Connecting arm, (**D**) Control chamber. (**B**) Response of *M*. *incognita* to root volatiles of three pepper cultivars and one accession compared to a control (moist sand). N corresponds to the total mean of responding J2 while n is the number of J2 corresponding to a given treatment. The level of significance is indicated by: ^*^P < 0.0001; ^#^P < 0.05; ns = not significant at P = 0.05. (**C**) A schematic representation of the volatile collection set-up in the laboratory. (**D**) Gas chromatography-mass spectrometry chromatograms of root volatiles of *Capsicum annum*. Numbers correspond to the following compounds (1) α-Pinene (2) Decane (3) D-Limonene (4) (Z)-β-Ocimene (5) *p*-Cymene (6) Undecane (7) Camphor (8) 2-Methoxy-3-(1-methylpropyl)-pyrazine (9) Dodecane (10) Methyl salicylate (11) Thymol (12) Tridecane (13) Tetradecane (14) γ - Himachalene (15) Allo-aromadendrene (16) α –Muurolene (17) 4,5-Di-epi-aristolochene (18) γ – Gurjunene (see also Table 1). Asterisk (*) indicates matrix interferences present in the control and impurities.

To determine the responses of J2s to the root volatiles of the different pepper cultivars, we used a dual choice soil olfactometer (Fig. 1a) to assay the preferences of J2s to pepper roots and moist sand (control) volatiles. A greater (P < 0.0001) number of nematodes preferred root volatiles from cv. California wonder (82%, χ^2^ = 60.06, df = 1), Yolo Wonder (74%, χ^2^ = 28.14, df = 1), and Long Red Cayenne (71%, χ^2^ = 60.06, df = 1) over the control. However, the control was more preferred than the root volatiles from AVDRC PP0237 (63%, χ^2^ = 5.94, df = 1, P = 0.01) (Fig. 1b).

### Coupled gas chromatography/mass spectrometric identification of *Capsicum annum* root volatiles

We used GC/MS analysis to identify 18 components represented by the peaks 1, 3, 4, 5, 7, and 11 (monoterpenoid), 8 (methoxypyrazine) 2, 6, 9, 12, and 13 (alkanes), 10 (ester) and 14–18 (sesquiterpenes) in the root volatiles of the four peppers (Fig. 1d). The identities of compounds 1, 3, 4, and 6 to 12 were confirmed by comparison of their retention times and mass spectral fragmentation with authentic standards. Compounds 2, 5, and 13 to 18 were tentatively identified based on mass spectral library data only. Of these, six components (α-pinene, limonene, 2-methoxy-3-(1-methylpropyl)-pyrazine, methyl salicylate, tridecane, and 4,5-di-epi-aristolochene), were each detected in the volatiles of all four peppers but to varying concentrations (Table 2). The sweet pepper cultivars differed in their volatile root chemistry, with more sesquiterpenes dominating the volatiles of cv. California Wonder than cv. Yolo Wonder, while the monoterpenes camphor and (*Z*)-β-ocimene were exclusively detected in cv. Yolo Wonder. Additionally, the two hot peppers Long Red cayenne and AVDRC PP0237 also showed similarity and variance, with the monoterpene thymol being specific to the AVDRC PP0237. The compound produced in highest quantity in accession AVDRC PP0237 and cv. Yolo Wonder was the sesquiterpene 4, 5-di-epi-aristolochene, with the alkane tetradecane in cv. Long Red cayenne, and the sesquiterpene γ - gurjunene in cv. California Wonder.

**Table 2:** Mean amount (pg/plant/hr ± SEM) of pepper root volatiles detected.

Peak #	RT (min)	Compound Name	Class of compound	Mean amount detected pg/plant/hr ± SEM			
				California Wonder	Yolo Wonder	Long Red Cayenne	AVDRC PP2037
1	9.76	α-Pinene^1^	Monoterpenoid	68.09 ± 34.10	47.72 ± 8.60	22.87 ± 4.56	21.90 ± 2.27
2	11.15	Decane	Alkane	25.41 ± 4.58	101.24 ± 9.87	—	—
3	11.7	D-limonene^1^	Monoterpenoid	61.24 ± 8.33	173.91 ± 33.59	80.38 ± 13.93	52.81 ± 16.22
4	11.87	(Z)-β-ocimene	Monoterpenoid	—	87.75 ± 18.54	—	—
5	12.15	p-Cymene	Cyclic hydrocarbon	—	—	17.97 ± 4.66	24.29 ± 5.23
6	12.93	Undecane	Alkane	18.64 ± 5.03	76.67 ± 16.58	12.47 ± 4.22	—
7	13.74	Camphor	Monoterpenoid	—	103.36 ± 24.58	—	—
8	14.13	2-Methoxy-3-(1-methylpropyl)-pyrazine^1^	Pyrazine	13.92 ± 4.31	20.70 ± 2.79	39.97 ± 7.82	13.85 ± 1.04
9	14.43	Dodecane	Alkane	25.52 ± 2.57	38.55 ± 3.97	—	48.42 ± 4.52
10	14.52	Methyl salicylate^1^	Ester	78.79 ± 7.91	57.86 ± 7.58	49.66 ± 4.72	48.29 ± 6.46
11	15.8	Thymol^2^	Monoterpenoid	—	—	—	48.43 ± 9.95
12	15.98	Tridecane^1^	Alkane	99.85 ± 5.18	172.45 ± 47.65	157.28 ± 13.73	75.56 ± 18.43
13	17.23	Tetradecane	Alkane	—	129.68 ± 30.77	284.71 ± 67.76	93.44 ± 36.68
14	18.13	γ - Himachalene	Sesquiterpene	68.37 ± 6.40	—	—	—
15	18.18	Allo-aromadendrene	Sesquiterpene	67.24 ± 7.62	—	—	—
16	18.31	Alpha-Muurolene	Sesquiterpene	115.10 ± 12.01	—	—	—
17	18.58	4,5-Di-epi-aristolochene1	Sesquiterpene	145.61 ± 31.89	231.18 ± 39.66	229.99 ± 39.33	234.44 ± 66.67
18	18.82	γ - Gurjunene	Sesquiterpene	553.83 ± 124.46	—	—	—

^1^Compounds common to the four pepper plants and ^2^compound specific to AVDRC PP0237.

### Differential responses of *Meloidogyne incognita* to natural root volatiles confirmed with synthetic chemicals

**Figure 2:**
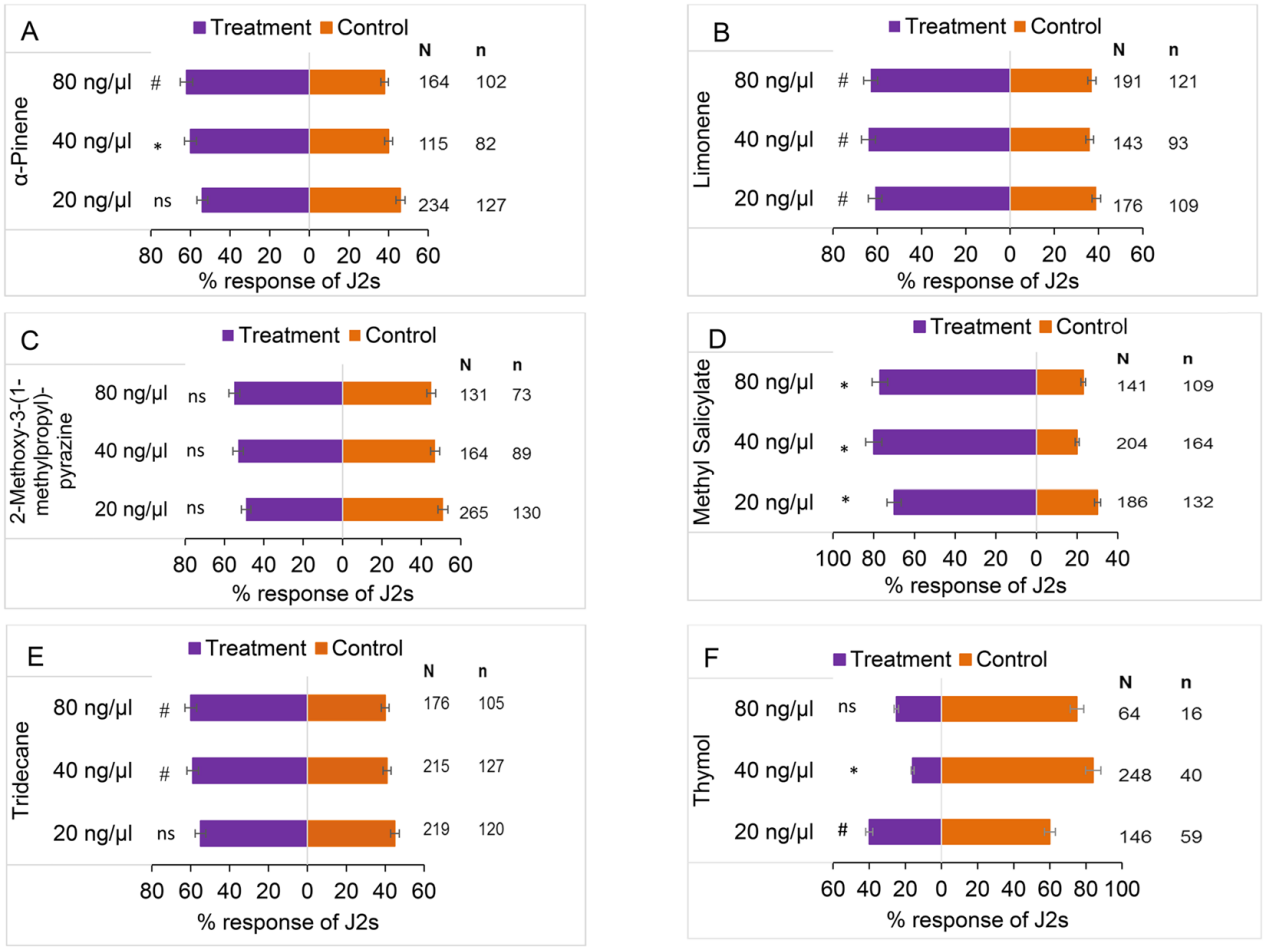
Differential responses of *Meloidogyne incognita* to individual synthetic compounds identified in pepper root volatiles. Response of *Meloidogyne incognita* infective juveniles (J2s) to different doses of (**A**) α-pinene compared to a control (moist sand). (**B**) Limonene compared to a control (**C**) 2-methoxy-3-(1-methylpropyl)pyrazine compared to a control (**D**) methyl salicylate compared to a control (**E**) tridecane compared to a control (**F**) thymol compared to a control. N corresponds to the total number of responding J2 while n is the number of J2 corresponding to a given treatment. The level of significance in indicated by ^*^P < 0.0001, ^#^P < 0.05 and ns = not significant at P = 0.05.

To determine the responses to the shared components, we tested J2 preference to five out of the six shared components because the sixth component, 4,5-di-epi-aristolochene, was not available (refer to methods section, bioassays with synthetic compounds). The J2s responded differentially to the individual compounds tested against a control in three doses. There was no difference in J2 response to α-pinene at 20 ng/µl (54%, χ^2^ = 1.54, df = 1; P = 0.21). J2s preferred α-pinene at 40 ng/µl (60%, χ^2^ = 18.64, df = 1, P = 1.57e-05) and 80 ng/µl (62%, χ^2^ = 9.27, df = 1, P = 0.0023) respectively compared to the control (Fig. 2a). Limonene was preferred at 20 ng/µl (61%, χ^2^ = 9.55, df = 1, P = 0.002), 40 ng/µl (64%, χ^2^ = 12.34, df = 1, P = 0.0004) and 80 ng/µl (63%, χ^2^ = 13.09, df = 1, P = 0.0003) compared to the control (Fig. 2b). There was no difference in the directional orientation of J2s to 2-methoxy-3-(1-methylpropyl)-pyrazine at 20 ng/µl (49%, χ^2^ = 0.06, df = 1, P = 0.8), 40 ng/µl (53%, χ^2^ = 1.03, df = 1, P = 0.3) and 80 ng/µl (55%, χ^2^ = 1.49, df = 1, P = 0.22) when compared to the control (Fig. 2c). Methyl salicylate (MeSA) was preferred at 20 ng/µl (70%, χ^2^ = 31.876, df = 1, P = 1.643e-08), 40 ng/µl (80%, χ^2^ = 74.162, df = 1, P = 2.2e-16) and 80 ng/µl (77%, χ^2^ = 31.876, df = 1, P = 1.55e-10) compared to the control (Fig. 2d). Methyl salicylate was the most attractive compound and the highest attractive dose recorded was used in further experiments. There was differential response of J2s to tridecane at the three doses (Fig. 2e). At 20 ng/µl, there was no difference (55%, χ^2^ = 1.83, df = 1, P = 0.18) in J2 response compared to the control. Nematodes preferred tridecane at 40 ng/µl (59%, χ^2^ = 6.72, df = 1, P = 0.0096) and 80 ng/µl (60%, χ^2^ = 6.19, df = 1, P = 0.013) compared to the control. Interestingly, J2 responses were reduced by thymol (Fig. 2f) at 20 ng/µl (60%, χ^2^ = 4.99, df = 1, P = 0.025) and 40 ng/µl (84%, χ^2^ = 27.11, df = 1, P < 0.0001). There was no difference in the directional orientation of J2s at 80 ng/µl (75%, χ^2^ = 3.06, df = 1, P = 0.08). Optimal reduction in J2 response was observed at a concentration of 40 ng/µl based on statistical analysis hence, this dose was used in further experiments.

**Figure 3:**
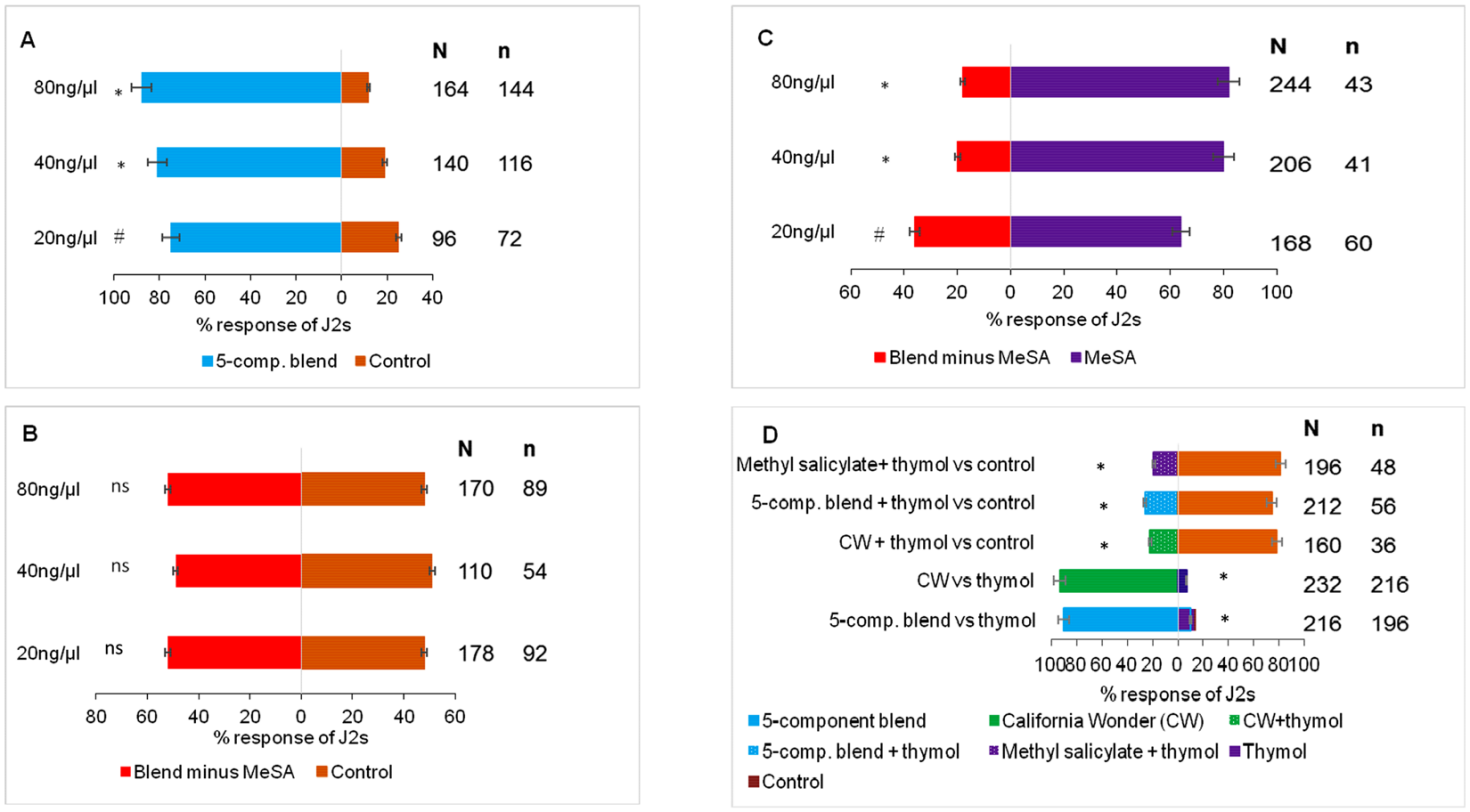
Differential responses of *Meloidogyne incognita* to synthetic blends and effect of thymol on their chemotaxis responses. Response of *Meloidogyne incognita* infective juveniles (J2s) to different doses of (**A**) 5-component blend compared to a control (moist sand). (**B**) Blend minus methyl salicylate (MeSA) compared to a control and (**C**) blend minus MeSA versus MeSA. N corresponds to the total number of responding J2 while n is the number of J2 corresponding to a given treatment. (**D**) Effect of thymol on response of *Meloidogyne incognita* infective juveniles (J2s) to methyl salicylate, the 5-component blend and cultivar California Wonder (CW). N corresponds to the number of responding J2 while n is the number of J2 corresponding to a given treatment. The level of significance in indicated by ^*^P < 0.0001, ^#^P < 0.05 and ns = not significant at P = 0.05.

In assays with blends, J2s preferred the three doses of the five-component synthetic blend compared to the control (Fig. 3a); at 20 ng/µl (71%, χ^2^ = 5.04, df = 1, P = 0.025), 40 ng/µl (81%, χ^2^ = 13.83, df = 1, P < 0.001) and 80 ng/µl (88%, χ^2^ = 21.95, P < 0.0001). A dose of 80 ng/µl was established as optimal based on statistical analysis and then used in further experiments.

In the experiment comparing J2 responses to the blend (alpha-pinene + limonene + 2-methoxy-3-(1-methylpropyl)-pyrazine + tridecane) without MeSA versus a control (Fig. 3b), there was no difference in J2 response at 20 ng/µl (52%, χ^2^ = 0.14, df = 1, P = 0.70), 40 ng/µl (81%, χ^2^ = 0.009, df = 1, P = 0.924) and 80 ng/µl (88%, χ^2^ = 0.288, df = 1, P = 0.59). J2s preferred MeSA in the experiment comparing their responses to the blend (alpha-pinene + limonene + 2-methoxy-3-(1-methylpropyl)-pyrazine + tridecane) without MeSA versus MeSA alone (Fig. 3c). J2s chose MeSA at 20 ng/µl (64%, χ^2^ = 13.149, df = 1, P = 0.0003), 40 ng/µl (80%, χ^2^ = 73.442, df = 1, P < 2.2e-16) and 80 ng/µl (82%, χ^2^ = 101.02, df = 1, P < 2.2e-16).

### Effect of thymol on natural plant volatiles and the preferred five-component blend

To determine the significance of thymol in RKN host location, we tested the response of J2s to thymol versus cultivar California Wonder and the five-component synthetic blend. J2s preferred volatiles of cv. California Wonder (92%, χ^2^ = 41.39, df = 1, P < 0.0001) and the five-component synthetic blend (90%, χ^2^ = 34.24, df = 1, P < 0.0001) relative to thymol (Fig. 3d). Interestingly J2 responses were significantly lower when cv. California Wonder intact root volatiles were spiked with 40 ng/µl of thymol (78%, χ^2^ = 11.03, df = 1, P < 0.001). A similar response was observed when 80 ng/µl of methyl salicylate (χ^2^ = 39.293, df = 1, P = 3.648e-10) and the five-component blend (74%, χ^2^ = 10.87, df = 1, P < 0.001) was spiked with 40 ng/µl of thymol respectively compared to the control (Fig. 3d).

## Discussion

Our RKN infection experiment, which showed that the sweet pepper cultivars, Yolo Wonder and California Wonder, exhibited higher galling and egg mass indices is consistent with previous reports that both are highly susceptible to RKNs^19, 33^. These indices were also high for the hot pepper Long Red Cayenne, whereas AVDRC PP0237 did not support the growth and multiplication of *M*. *incognita* suggesting that mechanical and chemical cues are involved in J2 host selection and discrimination. In addition, J2 host selection may be dependent on the race or strain of *M*. *incognita* which needs to be investigated. In the present study, we hypothesized that chemical cues may mediate host location by the infective juveniles. Results of our olfactometer assays confirmed our hypothesis, which were also in agreement with previous results reported by Prot^27^ that host roots may attract or repel plant-parasitic nematodes. However, Prot’s study did not identify the mediating host chemical signals. Nonetheless, these results suggest that *M*. *incognita* may have established a strong inclination for pepper plants that may best support its survival and multiplication, evidenced by its ability to distinguish host root volatiles even within species.

Chemical analyses identified complex blends of volatile compounds emitted by roots of the pepper cultivars belonging to several chemical classes. Interestingly, six of these compounds were produced by all the pepper plants although in different relative amounts: α-pinene, limonene, 2-methoxy-3-(1-methylpropyl)-pyrazine, methyl salicylate (MeSA), tridecane and 4,5-di-epi-aristolochene. Thymol was exclusively detected in the AVDRC PP0237 accession. In dose-response bioassays, the nematodes responded differently to the synthetic compounds. Individually, MeSA was the most effective in inducing positive chemotaxis. Its significance in J2 host location was confirmed in tests whereby subtraction of MeSA from the 5-component blend significantly reduced the attraction of J2s. Equally, thymol tested alone or tested in combination with the attractive pepper plant and synthetic chemical blend reduced J2s responses, suggesting that its presence in the root volatiles of AVDRC PP0237 accession may have contributed to the observed nematode responses. However, additional studies with other RKN-resistant pepper cultivars need to be undertaken to determine if thymol is indeed the antagonistic chemical component or whether other compounds may be involved across cultivars. It would also be important to investigate the role of the sesquiterpenes tentatively identified as 4,5-di-epi-aristolochene, common to the four pepper plants, and γ – gurjunene, the most abundant volatile released from the roots of cv. California Wonder, as well as tetradecane in order to determine their roles in J2 host location and discrimination. Future work should also investigate if *M*. *incognita* is attracted to a blend of γ –himachalene, allo-aromadendrene, α-muurolene and γ – gurjunene, the sesquiterpenes present in California Wonder and also compare the responses of *M*. *incognita* to the sesquiterpene blend versus thymol.

Some of the VOCs identified in the current study have been reported to mediate various above-ground plant-herbivore interactions in a number of solanaceous crops. For instance, α-pinene, limonene, γ – gurjunene and α –muurolene are components of the complex mixture of headspace volatiles of tomato involved in the host location and oviposition of the tomato leaf miner, *Tuta absoluta* (Lepidoptera: Gelechiidae)^35–37^. A monoterpene blend which included camphor, α-pinene and limonene in essential oils of the African nightshade, *Solanum sarrachoides* Sendtner contributed to oviposition deterrence against the tomato red spider mite, *Tetranychus evansi* Baker and Pritchard^38^. Conversely, in below ground chemical communication, α-pinene and limonene among other VOCs, were shown to be possible attractants for the plant-parasitic nematode, *T*. *semipenetrans* ^14^. Additional studies should isolate or synthesize the compounds that were tentatively identified in the pepper cultivars in order to investigate their role in the plant-RKN interactions.

Interestingly, MeSA is synthesized from salicylic acid (SA), a phytohormone that is involved in both local and systemic plant induced resistance defenses. Both of these compounds are key components in the shikimic acid pathway which plays an important role in direct and indirect plant defenses^39^. Methyl salicylate is produced by plants in response to pest attack, for instance in *Nicotiana attenuatta* (Solanaceae) following attack by the larvae of *Manduca quinquemaculata* (Lepidoptera, Sphingidae)^40^, as well as by tomato and *Datura wrightii* (Solanaceae) when damaged by *Manduca sexta* (Sphingidae)^41^. In addition, the production of MeSA attracts natural enemies of insect herbivores, for example, in attraction of the predatory mite *Phytoseiulus persimilis* Athias Henriot, when lima bean is infested with the two-spotted spider mite, *Tetranychus urticae* Koch^42^. In nematode interactions, several volatiles including MeSA were shown to influence positive chemotaxis of the EPNs *Heterorhabditis bacteriophora* Poinar and *Steinernema carpocapsae* (Rhabditidia: Steinernematidae)^43^. In the present study, we found that MeSA contributed significantly to the kairomonal signal in the host finding behavior of the *M*. *incognita* J2s.

On the other hand, thymol, occurs naturally as a biocide in plants such as *Thymus vulgaris* ^44^. It has antifungal^45^ and antibacterial activity^46^ in addition to nematicidal activity e.g. against the peanut root-knot nematode, *Meloidogyne arenaria* (Neal), the soybean cyst nematode, *Heterodera glycines* Ichinohe, the soil saprophytic nematode, *Caenorhabditis elegans* Maupas and the pinewood nematode, *Bursaphelenchus xylophilus* (Steiner and Buhrer) Nickle^47–49^. In our study, thymol demonstrated a potential role for its use in disrupting chemotactic host finding behavior of the motile and infective stage of RKNs. From a biosynthetic perspective, thymol is a phenolic monoterpene, a derivative of cymene. It has been established that γ-terpinene goes through aromatization to form *p-*cymene that is hydroxylated to thymol or its isomer carvacrol^50, 51^. In the current study, γ-terpinene was not detected in pepper root volatiles. However, *p-*cymene was detected in both hot pepper plants, Long Red Cayenne and AVDRC PP0237, but it was absent in the sweet peppers. This may indicate that genetic variations in biosynthetic pathways exist in plants of the same species. Therefore, a thorough genetic analysis of the four pepper cultivars should be initiated.

In summary, our results indicate that volatile chemical cues are important for *M*. *incognita* J2 to locate their preferred host, opening a promising possibility for the use of semiochemicals in the management of RKNs. These findings lay down some groundwork for exploring specific molecular pathways for suppressing root production of methyl salicylate or to incorporate genes responsible for thymol production in the roots of pepper for protection against RKN infection without altering other traits such as pepper flavor. A sustainable approach for delivering seed with resistant traits can be achieved through genetic engineering of secondary metabolite pathways that produce insecticidal compounds^52^. Previously, the Me1 and Me3 genes were shown to confer resistance to cultivars Charlestone Belle and Carolina Wonder pepper^19^. This was associated with disease incidence where resistant cultivars recorded low galling index and egg production^34^. Our study provides new insights towards linking molecular methods with biochemical processes for plant protection against these plant-parasitic nematodes.

## Materials and Methods

### Plants

For the study, two sweet pepper cultivars: ‘California Wonder’ (CW) and ‘Yolo Wonder’ (YW), one hot pepper cultivar, Long red Cayenne (LR) and one hot pepper accession, AVDRC PP0237 were selected. The cultivars CW, YW and LR are highly susceptible to *M*. *incognita*, while AVDRC PP0237 is resistant^19, 33^. Seeds of cv. CW and LR seeds were purchased from local agrochemical stores (Simlaw Seeds Company Limited, Nairobi, Kenya) and cv. YW from East Africa Seed Company, Nairobi, Kenya. Seed of accession AVDRC PP0237 was obtained from the World Vegetable Center through Kenyatta University, Nairobi, Kenya. Seeds were sown in a rectangular basin (67 cm long, 40 cm wide and 5 cm in depth) containing sterilized sand (autoclaved at 121 °C for 40 min) at the International Centre of Insect Physiology and Ecology (*icipe*) Duduville campus, Nairobi, (1°16′60″S; 36°49′0″E). Seedlings were transplanted in 2 L plastic pots (17 cm top diameter, 13 cm base diameter and 15 cm depth) with sterilized sand two weeks after germination. Plants were watered daily each morning and maintained in a screenhouse at 22 ± 1 °C and 60–70% relative humidity (RH). Plants of 3–6 weeks old were used for the experiments.

### Nutrient Solution

Nutrient solution to provide macro and micro-nutrients prepared as described by Lambert *et al*.^53^, was used. The stock solution contained autoclaved (121 °C) Ca(NO_3_)_2_.4H_2_O, 653 g/L; MgSO_4_.7H_2_O, 399 g/L; KNO_3_, 184 g/L and filter-sterilized (Whatmann filter paper, Grade 1, 27 cm diameter) NH_4_H_2_PO_4_, 108 g/L; FeSO_4_.7H_2_O 10 g plus 72 ml of 500 mM EDTA (pH 8.0) per liter and micronutrients (per liter; MnCl_2_.4H_2_O, 1.81 g; CuSO_4_.5H_2_O, 0.1 g; ZnSO_4_.5H_2_O, 0.22 g; H_3_BO_3_, 2.86 g; H_2_MoO_4_.H_2_O, 0.02 g). To formulate the amounts used for watering the plants, a 50 L plastic container (Kenpoly Manufacturers Limited, Nairobi, Kenya) was used and Ca(NO_3_)_2_, 25 ml; MgSO_4_, 25 ml; KNO_3_, 75 ml; NH_4_H_2_PO_4_, 25 ml; Fe/EDTA, 25 ml and micronutrients, 25 ml were mixed with distilled water to constitute a final volume of 50 L.

### Nematode population

Meloidogyne incognita were obtained originally from tomato (*Lycopersicon ensculentum*) in Taita Taveta County, Kenya (3.3161°S, 38.4850°E) and maintained thereafter in pure cultures on tomato cv. Cal J seedlings in pots containing sterilized sand in the screen house at *icipe*. Egg masses of *M*. *incognita* were extracted from infected roots under a stereomicroscope (Leica M125, Leica microsystems, USA) and placed in 12-well culture plates containing distilled water to allow hatching in the dark at 27 ± 2 °C for 2 to 5 days. The first juvenile stage (J1) emerges within the egg within 2 days^16^. After 2 days the emerged J2s were then counted with a hand tally counter under the stereomicroscope and they were transferred into 15 ml falcon tubes using a plastic transfer pipettes until it was used in the bioassays.

### Inoculation assays

This experiment was carried out to evaluate *M*. *incognita* infection of pepper using greenhouse screening technique described by Holdbrook *et al*.^54^, in four replicates. Each pot containing five plants was considered as a replicate. The plants were grown in 1 L plastic pots (10 cm diameter x 15 cm height) filled with sterilized sand and each pot was inoculated with 500 J2s. Approximately 45 days after inoculation, pepper plants were uprooted and rinsed free of soil. Roots were placed in 500 ml beakers containing 300 ml 1.5% Phloxin B solution for 20 min^55^ to stain the egg masses for ease of counting. Each plant was indexed for root galls and egg masses using the following scale: 0 = no galls or no egg masses, 1 = 1 to 2, 2 = 3 to 10, 3 = 11 to 30, 4 = 31 to 100, and 5 = more than 100 galls or more than 100 egg masses per plant^16, 22^.

### Dual choice olfactometer assays with intact plants

The response of J2s to root-produced volatiles was tested in a modified dual choice olfactometer (SigmaScientific, Gainesville, Florida)^10^. The olfactometer comprised of four components; the stimulus chamber (A) and the control chamber (D) both measuring 85 mm diameter × 140 mm depth with a connector (15 mm diameter x 30 mm long) fitted with an ultra- fine mesh screen. The central release arm (B) (20 mm diameter × 60 mm length) linked to detachable connecting arms (C) (20 mm diameter × 70 mm length) that connected chambers A and D (Fig. 1a). For the dual choice olfactometer assays, 30 plants were conditioned by placing in growth chambers containing 300 g of sterilized sand. They were watered with 20 ml of the nutrient solution daily for 3–5 days prior to the experiment in the laboratory and maintained at 25 ± 2 °C. In the control chamber, 300 g of sterilized sand was placed and 50 ml nutrient solution added. In the connection chamber, 30 g of sand was used and 20 ml nutrient solution added. Four replicates each comprising 500 juveniles were conducted. Each growth chamber containing 30 plants paired with a growth chamber containing 300 g moist sand (control) was considered as a replicate. After 4 hr (the optimal recovery time following preliminary studies testing response of J2 after 2, 6, 12, and 24 hr), the olfactometer was disassembled and the sand in each detachable section placed on a Baermann extractor^55^ for 24 hr to recover the J2s. A 27 µm mesh standard test sieve was used to collect the J2, which were stored in 50 ml falcon tubes. The number of J2s recovered from sections C (the connecting arms linking the stimulus and control chamber), A and D were counted using a hand tally counter under the stereo microscope at a magnification of 25x.

### Collection of root volatiles

Thirty pepper plants each of the cultivars or accession were pre-conditioned for volatile sampling in glass chambers for 3–5 days. Volatiles were collected for 24 hr on a pre-cleaned (dichloromethane and nitrogen dried) Super Q (30 mg, Analytical Research System, Gainesville, Florida, USA) adsorbent. Each adsorbent was connected to a steel probe (17 cm long, 0.5 cm i.d.) inserted in the plant sand root zone in the glass chamber. The probe was connected to a vacuum pump that extracted volatiles from the soil at 170 ml/min. Cleaned charcoal filters (activated charcoal) were used to cover the sand to prevent adsorption of other VOCs from the surrounding air. The Super Q filters were eluted with 200 μl of GC-grade dichloromethane (Sigma Aldrich, St. Louis, Missouri, USA) and concentrated to 50 μl under a stream of nitrogen to enable detection of compounds that are present in very trace amounts when carrying out GC-MS analysis. The experiment was carried out in triplicates to quantify the amounts of identified components in the root volatiles of pepper plants. Each growth chamber containing 30 plants was considered as a replicate. For the control, volatiles were collected similarly from 300 g of pre-conditioned sand.

### Analysis of root volatiles

Extracts of the root volatiles were analyzed using coupled gas chromatography-mass spectrometry (GC-MS) on an Agilent Technologies 7890B GC linked to a 5977 MS, equipped with a non-polar HP-5 MS ultra-inert column (30 m × 0.25 mm i.d., 0.25 μm) (J&W, Folsom, CA, USA). The temperature program was 5 min at 35 °C, then 10 °C/min to 280 °C. A 1 µl aliquot of each volatile extract was analyzed in the splitless mode using helium as a carrier gas at a flow rate of 1.2 ml/min. Spectra were recorded at 70 eV in the electron impact (EI) ionization mode. Compounds were identified by comparison of mass spectral data with library data: Adams2^56^ and NIST08^57^. Unambiguous structure assignments were based on co-injection with commercially available authentic standards. Quantification was based on calibration curves (peak area vs. concentration) generated from authentic standards of identified compounds.

### Chemicals

Authentic standards of (R)-(+) α-pinene (purity 99%), 2-methoxy-3-(1-methylpropyl)-pyrazine (purity 99%), thymol (99%), and tridecane (purity 99%) were purchased from Sigma Aldrich, St. Louis, MO, USA. Methyl salicylate (purity 97%) and (R)-(+)-limonene (purity 97%) were purchased from Sigma Aldrich, Steinhelm, Germany.

### Bioassays with synthetic compounds

To determine the role of the identified root VOCs in the host-seeking behavior of RKNs, the responses of J2s were assessed in fourteen treatments (Supplementary Table S1). Dual choice olfactometer was used in dose response assays consisting of: a) the common compounds identified in the pepper root volatiles either alone or in blends (mean of the naturally occurring proportions in pg/plant/hr: α-pinene, 40 pg/plant/hr; limonene, 92 pg/plant/hr; 2-methoxy-3-(1-methylpropyl)-pyrazine, 22 pg/plant/hr; methyl salicylate, 59 pg/plant/hr and tridecane, 126 pg/plant/hr); the sixth component was tentatively identified based on comparison of mass spectral data with library data only as the standard was not commercially available, hence was not included for bioassays; b) thymol, 48 pg/plant/hr, identified as specific to the roots of the accession AVDRC PP0237. Three concentrations of 20 ng/µl (the natural amount of thymol detected in the root volatiles of the resistant AVDRC PP0237, hence standardized across for comparison purposes), 40 ng/µl and 80 ng/µl (obtained by doubling the preceding concentration), each prepared in hexane. Hexane was used for bioassays with synthetic compounds because it was not toxic as dichloromethane which was used in chemical analysis. The treatments were applied by dispensing 2 ml aliquots into the stimulus chamber containing 300 g of sterilized sand. The control consisted of 2 ml solvent (hexane) dispensed in 300 g sterilized sand. Each dose was tested against a control and carried out in four replicates. The optimal doses were used for further bioassays; thymol (40 ng/µl) was tested against cv. California Wonder (the most preferred cultivar in our assays) and also paired with the optimal dose of the five-component synthetic blend at a concentration of 80 ng/µl. Another experiment assessed the effect of spiking methyl salicylate (80 ng/µl), the five-component synthetic blend (80 ng/µl) as well as cv. California Wonder with thymol (40 ng/µl) tested against a control.

### Statistical analysis

The mean number of galls and egg masses for scoring the galling and egg mass indices were analyzed using analysis of variance and means separated using Duncan’s multiple range test at P ≤ 0.05. The number of responding nematodes obtained from the dual choice olfactometer assays were recorded as means of J2 that responded to the different treatments and expressed as percent response [(n/N) × 100]. N corresponds to the total number of responding J2, while n is the number of J2 corresponding to a given treatment. Non-respondents were not included in the analysis. The data from the dual choice olfactometer assays was analyzed by Chi-square goodness of fit to assess (a) *M*. *incognita* discrimination to plant root volatiles compared to a control (sand); (b) attraction or avoidance of *M*. *incognita* to different doses of synthetic compounds tested individually or in blends against their respective controls. R version 2.15.1 software^58^ was used to perform the statistical analysis and all tests were performed at 5% significance level.

## Electronic supplementary material

Supplementary Table S1
