# Supplementary material for: Parasitic nematode Meloidogyne incognita interactions with different Capsicum annum cultivars reveal the chemical constituents modulating root herbivory
Source: Sci Rep. 2017 Jun 6;7:2903. doi: 10.1038/s41598-017-02379-8 (PMC5460232; doi:10.1038/s41598-017-02379-8)
Supplement: Supplementary file 1 — Supplementary Table S1 [file 41598_2017_2379_MOESM1_ESM.doc]

**Supplementary Information**

**Parasitic nematode** *Meloidogyne incognita* **interactions with different** *Capsicum annum* **cultivars reveal the chemical constituents modulating root herbivory**

**Ruth Kihika**^1, 2^, **Lucy K. Murungi**^3^, **Danny Coyne**^4^, **Margaret Ng’ang’a**^2^, **Ahmed Hassanali**^2^, **Peter E.A. Teal**^5†^ **and** **Baldwyn Torto**^1*^

^1^Behavioural and Chemical Ecology Unit, International Centre of Insect Physiology and Ecology, P.O. Box 30772-00100 Nairobi, Kenya

^2^Kenyatta University P.O. Box 43844- 00100 Nairobi, Kenya

^3^Jomo Kenyatta University of Agriculture and Technology, P.O Box 62,000 00200 Nairobi, Kenya

^4^International Institute of Tropical Agriculture (IITA), P.O. Box 30772-00100, Nairobi, Kenya

^5^USDA/ARS-CMAVE, 1600/1700 SW23rd Dr. Gainesville, FL 326

^†^ Peter Teal passed away on 11^th^ February 2015

***Correspondence:** [**btorto@icipe.org**](mailto:btorto@icipe.org)

|  | **Treatment comparisons using synthetic standards and blends** |
| --- | --- |
| **1** | α - Pinene versus sand |
| **2** | Limonene versus sand |
| **3** | 2-methoxy-3-(1-methylpropyl)-pyrazine versus sand |
| **4** | Methyl salicylate (MeSA) versus sand |
| **5** | Thymol versus sand |
| **6** | Tridecane versus sand |
| **7** | 5-component blend versus sand |
| **8** | Blend (α - pinene + limonene+ 2-methoxy-3-(1-methylpropyl)-pyrazine+ tridecane) minus MeSA versus sand |
| **9** | Blend (α - pinene + limonene+ 2-methoxy-3-(1-methylpropyl)-pyrazine+ tridecane) minus MeSA versus MeSA |
| **10** | 5-component blend + thymol versus sand |
| **11** | California Wonder + thymol versus sand |
| **12** | MeSA + thymol versus sand |

**Supplementary Table S1**: Treatment comparisons on response of J2 to synthetic compounds/blends
